# EpiFloripa COVID-Tel: methods and results of the telephone survey on the COVID-19 pandemic among older adults participating in the 2017–2019 and 2021–2022 EpiFloripa cohort study

**DOI:** 10.1590/S2237-96222026v35e20250021.en

**Published:** 2026-07-24

**Authors:** Eliana Funk, Danúbia Hillesheim, Thamara Hubler Figueiró, Ana Luisa Lages Belchor, Cassiano Ricardo Rech, Eleonora d’Orsi, Anna Quialheiro

**Affiliations:** 1 Universidade Federal de Santa Catarina Florianópolis Florianópolis SC Brazil Universidade Federal de Santa Catarina, Florianópolis, SC, Brazil; 2 Instituto Politécnico de Saúde do Norte Unidade de Investigação em Inteligência Artificial e Saúde Vila Nova de Famalicão BR Portugal Instituto Politécnico de Saúde do Norte, Unidade de Investigação em Inteligência Artificial e Saúde, Vila Nova de Famalicão, BR, Portugal

**Keywords:** COVID-19, Coronavirus Infections, Aged, Pandemics, Health Surveys, COVID-19, Infecciones por Coronavirus, Anciano, Pandemias, Encuestas Epidemiológicas

## Abstract

**Objectives:**

To analyze the impacts of the coronavirus pandemic on the health conditions and behaviors of older adults participating in the EpiFloripa Aging cohort study, comparing Wave 3 (2017–2019) with Wave 4 (2021–2022), and describe the methodology of the survey used.

**Methods:**

Telephone interviews were conducted. Data collection included sociodemographic and health information, adherence to distancing measures, internet use, and perceptions about the pandemic. Descriptive statistical analyses were performed to assess changes before and during the pandemic. Frequency measures (n, %) and 95% confidence intervals were calculated.

**Results:**

Data from 1,335older adults interviewed in Wave 3 and 354 participants in Wave 4 were used. There was an increase in internet use (from 48.5% in Wave 3 to 65.5% in Wave 4), indicating greater digital inclusion during the pandemic. Self-rated health showed that 55.6% maintained a positive perception, but 10.9% began to rate it negatively, and 61.4% began to present depressive symptoms. The majority of older adults (53.7%) rigorously followed social distancing measures; 23.2% reported worsening health. High adherence was observed for both the first (98.0%) and second vaccine doses (98.8%). However, 20.6% showed probable cognitive decline during the COVID-19 period.

**Conclusion:**

There was an increase in digital inclusion, adherence to distancing measures, and vaccination. A worsening in self-perceived health, increased cognitive decline, and depressive symptoms were observed. These findings highlight the need for public policies targeting mental health, digital inclusion, and social support for older adults in crisis contexts..

## Resumen

**Objetivos:**

Analizar los impactos de la pandemia de coronavirus en las condiciones de salud y los comportamientos de las personas mayores participantes de la cohorte EpiFloripa Anciano, comparando las olas 3 (2017-2019) y 4 (2021-2022), y describir la metodología de la encuesta utilizada.

**Métodos:**

Se realizaron entrevistas telefónicas. La recolección incluyó información sociodemográfica, de salud, adherencia a las medidas de distanciamiento, uso de internet y percepción sobre la pandemia. Se realizaron análisis estadísticos descriptivos para evaluar los cambios antes y durante la pandemia. Se calcularon medidas de frecuencia (n, %) e intervalos de confianza del 95%.

**Resultados:**

Se utilizaron datos de 1.335 personas mayores entrevistadas en la ola 3 y 354 participantes en la ola 4. Hubo un aumento en el uso de internet (del 48,5% en la ola 3 al 65,5% en la ola 4), lo que indicó una mayor inclusión digital durante la pandemia. La autoevaluación de salud mostró que el 55,6% mantuvo una percepción positiva, mientras que el 10,9% pasó a evaluarla negativamente y el 61,4% comenzó a presentar síntomas depresivos. La mayoría de las personas mayores (53,7%) siguió estrictamente las medidas de distanciamiento social; el 23,2% reportó un empeoramiento de su salud. Se observó una alta adherencia tanto a la primera dosis (98,0%) como a la segunda dosis (98,8%) de la vacuna. Sin embargo, el 20,6% presentó un probable declive cognitivo durante el período de la COVID-19.

**Conclusión:**

Hubo un aumento de la inclusión digital y de la adherencia a las medidas de distanciamiento y vacunación. Se observó un empeoramiento de la percepción de la salud, un incremento del declive cognitivo y de los síntomas depresivos. Estos hallazgos destacaron la necesidad de políticas públicas dirigidas a la salud mental, a la inclusión digital y al apoyo social para las personas mayores en contextos de crisis.

**Ethical aspects**

This research respected ethical principles, having obtained the following approval data:

**Research ethics committee** **Opinion number**, **Approval date**

Universidade Federal de Santa Catarina 1,957, 977, 9/3/2017

Universidade Federal de Santa Catarina 4,909,585, 16/8/2021

**Certificate of submission for ethical appraisal** 16731313.0.0000.0121, 48422821.5.0000.0121

**Informed consent form** Signed by all participants before the interview., For the telephone interviews, consent was obtained verbally.

## Introduction

The coronavirus pandemic, officially declared by the World Health Organization (WHO) in March 2020, posed an unprecedented challenge to global public health, disproportionately affecting older adults due to their greater vulnerability to severe health outcomes (1). Beyond its direct impact on physical health, the pandemic resulted in significant psychological and social consequences for older adults and aggravated social isolation, loneliness, and the risk of mental health disorders, such as depression and anxiety (2).

Analyzing the pandemic’s consequences for older adults is crucial for understanding its adverse effects and for developing mitigation strategies for future crises. In Canada and South Korea, more than 40% of older adults reported a worsening in self-perceived health (3,4). In Brazil, older adults experienced the greatest increase in feelings of sadness and fear, due to the risk of infection and social isolation (5).

Social distancing, essential to contain the virus, isolated older adults from support networks, affecting psychological well-being and hindering access to medical care and chronic disease management (1). The disruption of health services worsened hypertension and diabetes, increasing the risk of complications and hospitalizations (6).

In Brazil, the pandemic’s effects on older adults were exacerbated by social and economic inequalities. In many regions, limited access to healthcare and a high prevalence of comorbidities increased the risk of complications and deaths from coronavirus among this population (2). Moreover, the socioeconomic vulnerability of many Brazilian older adults, who rely on social programs such as the Continuous Cash Benefit (Benefício de Prestação Continuada, BPC) and retirement pensions, made this population even more susceptible to the economic effects of the pandemic, such as loss of household income and increased health-related costs (7).

Longitudinal studies, such as EpiFloripa Aging (*EpiFloripa Idoso*), are essential to understanding the pandemic’s impact on the lives of older adults, as they allow monitoring of the evolution of physical, mental, and social health over time. In Brazil, the lack of high-quality longitudinal data hampers the formulation of effective public health policies for older adults (8).

Given these challenges, analyzing the pandemic’s impact on the health of older adults is a priority for researchers, healthcare professionals, and policymakers. Understanding the effects on physical and mental health, access to services, and social well-being is essential for developing interventions and policies that promote healthy and resilient aging in the post-pandemic period (1).

This study aimed to analyze the impacts of the coronavirus pandemic on the health conditions and behaviors of older adults participating in the EpiFloripa Aginig cohort study, comparing Wave 3 (2017–2019) and Wave 4 (2021–2022), and to describe the methodology of the telephone survey conducted during the pandemic.

## Methods

### Study design 

This is a longitudinal study using data from the EpiFloripa Aging cohort. This was a population-based cohort conducted in Florianópolis, Santa Catarina, among older adults aged 60 years or older.

### Setting and participants

The EpiFloripa Aging cohort study started in 2009 with residents of the urban area of Florianópolis, aiming to investigate the living conditions and health of the older adult population over time. Data collection was conducted in waves, with home interviews in each wave: the first in 2009–2010, the second in 2013–2014, and the third in 2017–2019. During the pandemic, the fourth wave (2021–2022) was conducted, and its data were used in this study. Study details are available at: https://epifloripaidoso.paginas.ufsc.br/.

The third wave, conducted between 2017 and 2019, had its sampling plan, operational procedures for home interviews, and methodological strategies previously documented (9). Wave 4, named EpiFloripa COVID-Tel, was conducted during the pandemic via telephone interviews and aimed to assess the impacts of COVID-19 on the health, living conditions, and behaviors of this older adult population. The longitudinal design of the study enabled comparison of data collected before (Wave 3) and during (Wave 4) the pandemic.

In Wave 4, all Wave 3 participants were included, including those still under follow-up and those classified as losses or refusals. Between November 2021 and November 2022, telephone contact attempts were made to all eligible participants in Wave 3, the data collection period. 

Exclusion criteria for EpiFloripa COVID-Tel included: losses to follow-up and refusals among participants from the EpiFloripa Adults (*EpiFloripa Adulto*) cohort study who were invited to participate in the third wave; and newly selected participants who refused home interviews at this stage, thereby not providing information for this study. The participant, who, due to cognitive or auditory impairment, could not answer the questionnaire by telephone, may authorize an informant to provide answers on his behalf for specific sections of the interview.

Remote interviews enabled follow-up with older adults, who are vulnerable to coronavirus, without the risk of infection. This adaptation assessed the effects of social distancing on health outcomes. The method followed the Brazilian Longitudinal Study of Adult Health (*Estudo Longitudinal de Saúde do Adulto*, ELSA-Brasil), using adapted interviews and questionnaires to ensure study continuity (8).

### EpiFloripa COVID-Tel telephone interview 

Interviewers were recruited from undergraduate and graduate health programs at the Federal University of Santa Catarina (*Universidade Federal de Santa Catarina*, UFSC). After recruitment, training on the project and practice calls were conducted. All interviewers were instructed on the call workflow and received a manual. Eligible interviewers received contact lists of older adults for scheduling and conducting interviews.

Eligible older adults were contacted via landline or mobile phone, or through friends and family listed in the EpiFloripa Aging database. Interviewers identified themselves, and after oral consent, the interview proceeded. If the older adult was unavailable, the call was rescheduled.

The study questionnaire collected sociodemographic information and pandemic-related factors, including adherence to physical distancing guidelines, mask use, vaccination, positive COVID-19 test results, and hospitalization for COVID-19. All data collection instruments used in the survey consisted of previously validated questionnaires commonly employed in epidemiological studies, thereby ensuring the reliability and validity of the measures assessed. The complete questionnaire is available at: https://epifloripaidoso.paginas.ufsc.br/.

The questionnaire was transcribed using Research Electronic Data Capture (REDCap) v9.1.0. Quality control was performed through telephone calls to a 10% subsample of participants.

### Losses, refusals, and deaths

Deaths were identified during telephone calls. When a death was reported, information on the date, cause, and place of death was requested for database registration. Refusals were expressed verbally, whereas losses were defined after three contact attempts on different days and at different times, including weekends.

### Variables and measurement

This study used the following variables: sex (male, female), age group (60–69, 70–79, 80 years or older), years of schooling (1–4, 5–8, 9–11, 12 years or more), marital status (married/living with a partner, single, divorced, widowed), caregiver (yes, no), per capita income (≤1.0; 1.1–5.0; 5.1–10.0; ≥10.0 minimum wages), and internet use (yes, no).

Cognitive impairment was assessed using the Mini-Mental State Examination (*Mini Exame do Estado Mental*, MEEM) in Wave 3, classified according to Almeida (10), and the Montreal Cognitive Assessment (MoCA) (11), adapted for telephone administration, in Wave 4, categorized as no cognitive deficit or probable impairment. The scoring proposed by Trzepacz (12) was used to equate the instruments. Physical activity was measured using the International Physical Activity Questionnaire (IPAQ) (13), which is validated for older adults, and classified as inactive (0–9 min/week), insufficiently active (10–149 min/week), or active (≥150 min/week). 

Activities of daily living were evaluated in both waves using the Brazilian Multidimensional Functional Assessment Questionnaire (14), which was categorized as independent, mildly dependent, or moderately or severely dependent. Self-rated health was classified as positive or negative. Suspected depression was assessed using the Geriatric Depression Scale (GDS) (15), categorized as without depressive symptoms and with depressive symptoms, according to Almeida (15). 

Pandemic-related variables consisted of nine questions. The variable “During the first six months of the pandemic, how did you respond to the restrictions?” was nominal categorical, with options: “did nothing,” “took precautions but continued going out,” “stayed home, going out only for shopping,” and “stayed strictly at home.” The study included variables on self-perceived health (“stayed the same,” “improved,” “worsened slightly,” “worsened significantly”), changes in income (“increased,” “stayed the same,” “decreased slightly,” “decreased significantly,” “no income”), and frequency of use of public spaces (“never,” “every day,” “2–3 days per week,” “once per week,” “2–3 times per month,” “once per month”). Hospitalization, COVID-19 testing, vaccination (1st and 2nd doses), and pharmacy use were evaluated as dichotomous variables (yes/no).

### Statistical methods

Data were analyzed using descriptive statistics to characterize the sample of older adults from Waves 3 and 4, applying the “prop” command to adjust for sampling bias and 95% confidence intervals. Absolute and relative frequencies of the data were presented. 

To present change variables, new polytomous variables were created from dichotomous variables at Waves 3 and 4. These new variables were coded as: 1=continued to have the condition (1–1); 2=ceased to have the condition (1–0); 3=continued not to have the condition (0–0); and 4=began to have the condition (0–1). 

Analyses were performed using Stata, version 18.5, MP-Parallel edition.

## Results

The results presented refer to two distinct periods of the study: Wave 3, conducted between 2017 and 2019, in which 1,335 older adults were interviewed in person; and Wave 4, conducted between 2021 and 2022. For Wave 4 implementation, eligible individuals from previous waves were identified, totaling 1,646 older adults. Among these, 363 participated in the EpiFloripa COVID-Tel study (Wave 4), including 354 complete interviews and nine incomplete interviews—the latter interrupted at the participant’s request during the telephone call. Therefore, 354 completed telephone interviews were considered valid, representing 21.5% of the total eligible individuals. Losses to follow-up occurred due to deaths (n=74), refusals (n=274), and unsuccessful telephone contacts after three attempts or invalid numbers (n=935) (Figure 1).

**Figure 1:**
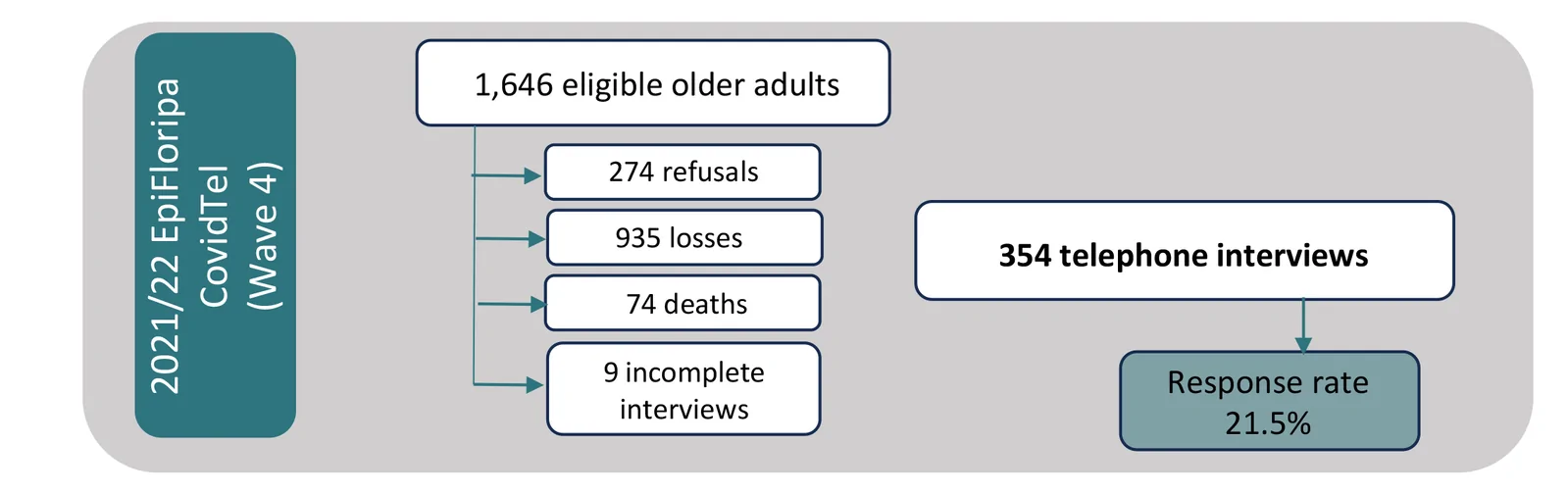
Wave 4 of the EpiFloripa Aging cohort study. Florianópolis, 2021–2022 (n=354)

The sociodemographic and health characteristics of older adults in Wave 3 and Wave 4 of the study are presented (Table 1). The sample was more female and older, with a higher proportion of adults aged 80 years and older in Wave 4 (31.0%). There was an increase in internet use among older adults, rising from 48.5% to 65.5%, as well as an increase in the proportion of those with probable cognitive decline (20.6%). Physical inactivity remained high (47.4%). A higher level of education was also observed: 42.0% of older adults had 12 or more years of schooling, and family income remained stable over the period analyzed (Table 1).

**Table 1:** Relative frequency (%) and 95% confidence interval (95%CI), according to sociodemographic characteristics: Wave 3 (pre–COVID-19) and Wave 4 (during COVID-19). Florianópolis, 2017–2022

Variable	Wave 3 (n=1,335) % (95%CI)	Wave 4 (n=354) % (95%CI)
Sex		
Female	61.0 (58.3; 63.6)	64.1 (58.9; 68.9)
Male	39.0 (36.4; 41.7)	35.9 (31.0; 41.0)
**Age group **(years)		
60–69	34.6 (32.0; 37.1)	18.4 (14.7; 22.8)
70–79	41.5 (38.9; 44.2)	50.6 (45.4; 55.8)
80+	23.9 (21.8; 26.3)	31.0 (26.5; 36.1)
**Schooling **(years)		
No formal education	4.7 (3.7; 5.9)	1.9 (0.8; 4.0)
1–4	28.8 (26.4; 31.3)	21.6 (17.5; 26.4)
5–8	17.0 (15.1; 19.1)	16.8 (13.2; 21.2)
9–11	17.1 (15.2; 19.3)	17.7 (13.9; 22.2)
12+	32.4 (29.9; 34.9)	42.0 (36.8; 47.4)
**Marital status**		
Married/living with a partner	55.4 (52.7; 58.0)	53.4 (48.2; 58.5)
Single	6.6 (5.4; 8.1)	4.8 (2.8; 7.3)
Divorced/separated	11.5 (9.9; 13.4)	7.1 (4.8; 10.3)
Widowed	26.5 (24.2; 29.0)	34.7 (30.7; 38.9)
Caregiver		
Yes	10.0 (8.5; 11.8)	9.1 (6.2; 12.2)
No	90.0 (88.2; 91.8)	90.9 (87.5; 93.5)
**Per capita income **(**minimum wage**)		
≤1,0	25.5 (23.2; 27.9)	21.6 (17.5; 26.4)
1,1–5,0	57.5 (54.8; 60.1)	57.4 (51.9; 62.6)
5,1–10,0	12.4 (10.8; 14.3)	15.0 (11.6; 19.3)
10,0+	4.6 (3.6; 5.8)	6.0 (3.9; 9.1)
**Internet use**		
Yes	48.5 (45.9; 51.2)	65.5 (60.4; 70.3)
No	51.5 (48.8; 54.1)	34.5 (29.7; 39.6)
**Cognitive decline**		
No cognitive decline	80.5 (78.3; 82.5)	79.4 (74.8; 83.3)
Probable cognitive decline	19.5 (17.5; 21.7)	20.6 (16.7; 25.2)
**Physical activity **(minutes/week)		
Inactive (0–9)	54.5 (51.8; 57.2)	47.4 (42.3; 52.7)
Insufficiently active (10–149)	18.6 (16.6; 20.8)	22.7 (18.6; 27.4)
Active (≥150)	26.9 (24.6; 29.3)	29.9 (25.3; 34.8)
**^B^OMFAQa **(ADL)^b^		
Independent	30.8 (28.3; 33.3)	41.2 (36.1; 46.5)
Mildly dependent	35.9 (33.4; 38.6)	27.4 (22.9; 32.3)
Moderately or severely dependent	33.3 (30.8; 35.9)	31.4 (26.7; 36.5)
**Self-rated health**		
Positive	61.9 (59.3; 64.6)	66.7 (61.2; 71.7)
Negative	38.1 (35.4; 40.7)	33.3 (28.3; 38.8)

^a^BOMFAQ: Brazilian Older Americans Resources and Services Multidimensional Function Assessment Questionnaire; ^b^ADL: activities of daily living.

Social distancing measures were strictly adhered to by 53.7% of participants, and 1.1% reported hospitalization for COVID-19. Perceived worsening of health was reported by 23.2% (slightly or significantly), and use of public spaces such as squares and parks was reduced (65.8% never attended). The data indicated high vaccination adherence, with 98.8% of participants having received the two recommended doses (Table 2).

**Table 2:** Absolute (n) and relative (%) frequencies for questions on older adults’ behaviors and their consequences during COVID-19 (Wave 4). Florianópolis, 2021–2022 (n=354)

Variable	Wave 4 n (%)
**During the first six months of the pandemic, how did you respond to the restrictions**?	
Did nothing, continued life as usual	12 (3.4)
Took precautions, kept distance from others, did not visit others, but continued working and/or going out	49 (13.8)
Stayed at home, going out only for shopping	101 (28.5)
Stayed strictly at home, leaving only for healthcare needs	190 (53.7)
Don’t know/Prefer not to answer	2 (0.6)
**During the pandemic, did you need to go out to the pharmacy**?	
Yes	46 (13.0)
No	306 (86.4)
Don’t know/Prefer not to answer	2 (0.6)
**Do you think the pandemic caused changes in your health status**?	
Stayed the same	263 (74.3)
Improved	5 (1.4)
Worsened slightly	59 (16.7)
Worsened significantly	23 (6.5)
Don’t know/Prefer not to answer	4 (1.1)
**In the past three months, during the pandemic, how often did you use any park, square, or beachfront near your home**?	
Never	233 (65.8)
Every day of the week	12 (3.4)
2–3 days a week	44 (12.4)
Once per week	18 (5.1)
2–3 times a week	15 (4.2)
Once per month	20 (5.7)
Don’t know/Prefer not to answer	12 (3.4)
**Did you get tested to find out if you were infected with the COVID-19 virus**?	
Yes	149 (42.1)
No	205 (57.9)
**Did you need hospitalization due to COVID**-19?	
Yes	4 (1.1)
No	350 (98.9)
**How did the pandemic affect your family’s income**?	
Increased	7 (2.0)
Stayed the same	263 (74.3)
Decreased slightly	53 (15.0)
Decreased significantly	23 (6.5)
No income	1 (0.2)
Don’t know/Prefer not to answer	7 (2.0)
**Did you receive the first dose of the COVID-19 vaccine**?	
Yes	347 (98.0)
No	7 (2.0)
**Did you receive the second dose of the COVID-19 vaccine**?	
Yes	343 (98.8)
No	3 (0.9)
Not applicable, received a single dose	1 (0.3)

Changes in health and functional conditions between Wave 3 and Wave 4 are highlighted (Figure 2). It was observed that 13.5% of older adults developed cognitive decline and 61.4% developed depressive symptoms during the pandemic. Self-rated health showed that 55.6% maintained a positive perception (Figure 2).

**Figure 2:**
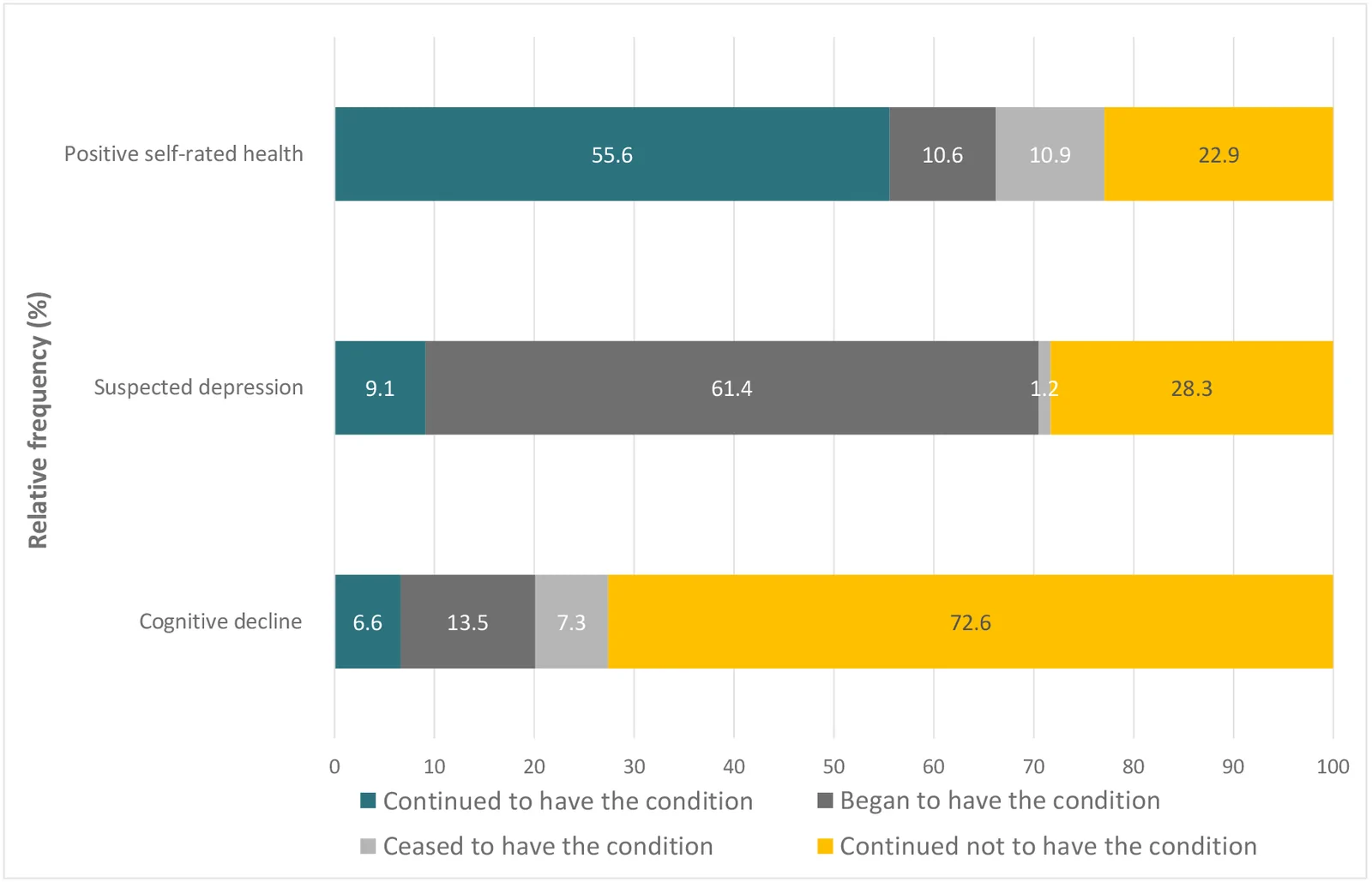
Relative frequency (%) of changes in health outcomes between Wave 3 (2017–2019) and Wave 4 (2021–2022). Florianópolis, 2017–2022 (n=354)

## Discussion

The results of this study highlighted the principal impacts of the COVID-19 pandemic on the health and behaviors of older adults in the EpiFloripa cohort. Overall, although many participants maintained positive health perceptions and adopted preventive measures, significant adverse effects were observed across cognitive, emotional, and physical domains. Digital inclusion emerged as an adaptive factor during social distancing, whereas high vaccination adherence reflected trust in public health measures.

Regarding cohort aging, the higher proportion of participants aged 80 years or older in Wave 4 was consistent with expectations in longitudinal studies, reflecting differential mortality and changes in age composition over time (8). The higher proportion of widowed participants in Wave 4 (34%) compared with Wave 3 (26%) corroborated the literature associating aging with increased widowhood, especially among women, and its effects on emotional well-being (16,17).

In addition to the absolute increase in internet use prevalence (from 48% to 65%), the data suggested advances in digital inclusion among older adults over the study period, possibly driven by increased demand for communication, information, and services during the pandemic. The use of digital technologies facilitated access to information, health services, and remote social interactions during the pandemic (18,19). However, on average, 34% of older adults remained without internet access, which may be related to socioeconomic and educational barriers (20). These technological inequalities indicated the need for public policies focused on digital literacy for older adults (21,22).

The results also revealed an increase in the prevalence of probable cognitive impairment (20%) and depressive symptoms (61%). The impact of social isolation, disruption of routines, and lack of cognitive stimulation are among the main factors associated with this decline (23). International studies corroborated these findings: in Italy, 30% of older adults reported worsening memory and attention (24); in the United States, approximately 24% of older adults reported subjective worsening of cognitive function during the pandemic, in a context marked by changes in routine and reduced engagement in cognitively stimulating activities (25); in Latin America, between 20% and 25% of older adults presented cognitive impairment, with higher prevalence among those with lower educational levels (6).

Regarding mental health, 61.4% of participants presented suspected depression in wave 4, an alarming result when compared with pre-pandemic periods. Feelings such as loneliness, grief, and fear of infection were identified as the main triggers for worsening of symptoms (3,17). In Spain, anxiety and depression increased in 40% of older adults during the pandemic (23), while in Brazil, there was also a high prevalence of psychological distress among older adults (2,5).

A marked reduction in physical activity was observed: 47% of older adults were classified as inactive and 22% as insufficiently active. This finding is consistent with evidence from Canada and Brazil, which indicated reductions of up to 60% in physical activity among older adults due to the closure of public spaces and the adoption of social distancing measures (20,21). Such a reduction contributed to a worsening of functional health and an increased risk of chronic diseases (21).

Negative self-rated health, reported by 10% of participants, was associated with social isolation and activity restrictions. Similar findings were observed in a study conducted in Korea (4), in which 20% of the 2,097 participants reported worsening health status after the pandemic, and in Taiwan (26), where negative perceptions of subjective well-being during the pandemic were correlated with poorer self-rated health among older adults. On the other hand, some older adults reported improvements or maintenance of positive health perceptions, suggesting the influence of factors such as the absence of severe disease, family support, and a sense of relative well-being compared to others’ experiences (26).

Regarding adherence to sanitary measures, 53% of older adults remained strictly at home, a pattern observed in Italy and France (25,27). This adherence contributed to the low hospitalization rate (1%) observed in Wave 4. Most participants also avoided visits to pharmacies (86%), indicating adaptation to delivery services and support from close social networks (27).

The stability of family income reported by 74% of participants may be related to the local socioeconomic structure, the high Human Development Index (HDI) of Florianópolis (0.847), and the presence of stable retirement benefits (28). This contrasted with studies conducted in less advantaged regions, where the pandemic had a greater economic impact on older adults (7,29).

Vaccination adherence was high: 98% of older adults received the first dose, and 98% of those who received the first dose also received the second. The findings reflected the success of COVID-19 immunization campaigns among older adults in Brazil, confirmed by evidence from another national study in which, among 4,364 participants, 91% intended to be vaccinated or had already been vaccinated (30).

This study presented relevant limitations. The use of self-report may introduce recall and response bias, particularly in subjective variables. The Wave 4 subsample, comprising 354 participants, is not representative of the original cohort study, and survival bias may have influenced the results, given that healthier older adults were more likely to remain in the sample. The loss rate between waves is comparable to that reported in other longitudinal studies involving older adults (6,8), but it limits the inferences that can be generalized. In addition, telephone-based data collection, although necessary, may have led to increased refusals due to mistrust of this approach (7).

Despite these limitations, they were minimized through methodological strategies such as interviewer training, the use of previously validated instruments adapted for telephone administration, the use of standardized manuals, and the implementation of quality control by repeating administration in a subsample. This ensured greater reliability of the collected data. 

The results of the EpiFloripa cohort showed that, although older adults adopted relevant adaptive strategies during the pandemic, adverse effects on mental, cognitive, and physical health were substantial. Such evidence reinforces the need for continuous and targeted public policies for older adults during health crises.

This study is among the few longitudinal analyses conducted in Brazil on the pandemic’s impacts on the older adult population. It provided valuable evidence to support intersectoral policies aimed at promoting digital inclusion, mental health support, and continuous access to healthcare, with a focus on fostering healthier aging.

## Data Availability

The database and analysis codes used in this study will be made available upon request by the reviewers.

## References

[1] Brooke J, Jackson D. Older people and COVID-19: Isolation, risk and ageism. J Clin Nurs. 2020 [cited 2024 Nov 1]. Available from: https://doi.org/10.1111/jocn.15274. 10.1111/jocn.15274 32239784

[2] Fundação Oswaldo Cruz. Saúde mental dos idosos durante a pandemia de covid-19 [Internet]. Rio de Janeiro: Fiocruz; 2021 [cited 2024 Nov 5]. Available from: https://portal.fiocruz.br/documento/saude-mental-dos-idosos-durante-pandemia-de-covid-19.

[3] Raina P, Wolfson C, Griffith L, Kirkland S, McMillan J, Basta N. A longitudinal analysis of the impact of the COVID-19 pandemic on the mental health of middle-aged and older adults from the Canadian Longitudinal Study on Aging. Nat Aging. 2021 [cited 2024 Nov 8]. Available from: https://doi.org/10.1038/s43587-021-00128-1. 10.1038/s43587-021-00128-1 37117519

[4] Kang E, Lee H, Sohn JH, Yun J, Lee JY, Hong YC. Impact of the COVID-19 Pandemic on the Health Status and Behaviors of Adults in Korea: National Cross-sectional Web-Based Self-report Survey. JMIR Public Health Surveill. 2021 [cited 2024 Nov 11]. Available from: https://doi.org/10.2196/31635. 10.2196/31635 PMC8629347 34653017

[5] Instituto Brasileiro de Geografia e Estatística. Pesquisa Nacional de Saúde 2020: impacto da covid-19 na saúde dos idosos [Internet]. Rio de Janeiro: IBGE; 2020 [cited 2024 Nov 15]. Available from: https://www.ibge.gov.br/estatisticas/sociais/saude/9160-pesquisa-nacional-de-saude.html.

[6] Wong R, Palloni A, Wong G, Mendoza-Alvarado LA. Health, Aging, and Retirement in Latin America and the Caribbean: An Overview of the SABE-LAC Study [Internet]. Washington: Inter-American Development Bank; 2019 [cited 2024 Nov 22]. Available from: https://publications.iadb.org/publications/portuguese/document/Envelhecer-na-Am%C3%A9rica-Latina-e-Caribe-prote%C3%A7%C3%A3o-social-e-qualidade-de-vida-entre-pessoas-idosas.pdf.

[7] Oliveira RM, Barbosa IM, Silva LAF. Impacto da pandemia de COVID-19 na participação de idosos em entrevistas remotas. Rev Bras Geriatr Gerontol. 2021 [cited 2024 Nov 28]. Available from: https://doi.org/10.1590/1981-22562021024.210086.

[8] Lima-Costa MF, Macinko J, Andrade FB, Souza Júnior PRB, Vasconcellos MTL, Oliveira CM. ELSI-COVID-19 initiative: methodology of the telephone survey on coronavirus in the Brazilian Longitudinal Study of Aging. Cad Saúde Pública. 2020 [cited 2024 Dec 5]. Available from: https://doi.org/10.1590/0102-311X00183120. 10.1590/0102-311X00183120 33053061

[9] Confortin SC, Figueiró TH, Marques LP, Medeiros PA, Ceolin G, D’Orsi E. Estudo de Coorte Epifloripa Idoso: abordagens metodológicas e reposição da amostra durante a onda 3 (2017-19). Estud Interdisc Envelhec. 2023 [cited 2024 Dec 10]. Available from: https://doi.org/10.22456/2316-2171.120481.

[10] Almeida OP. Mini Exame do Estado Mental e o diagnóstico de demência no Brasil. Arq Neuropsiquiatr. 1998 [cited 2024 Dec 12]. Available from: https://doi.org/10.1590/S0004-282X1998000400014 10.1590/s0004-282x1998000400014 9850757

[11] Nascimento LF, Nascimento DA. Avaliação Cognitiva de Montreal (MoCA): adaptação e validação para o Brasil. J Bras Psiquiatr. 2020 [cited 2024 Dec 14]. Available from: https://doi.org/10.1016/j.jbp.2019.06.001.

[12] Trzepacz PT, Hochstetler H, Wang S, Walker B, Saykin AJ. Alzheimer’s Disease Neuroimaging Initiative. Relationship between the Montreal Cognitive Assessment and Mini-Mental State Examination for assessment of mild cognitive impairment in older adults. BMC Geriatr. 2015 [cited 2024 Dec 16]. Available from: https://doi.org/10.1186/s12877-015-0103-3. 10.1186/s12877-015-0103-3 PMC4562190 26346644

[13] Böhning D, Vandelanotte C, Duncan MJ, Short CE. Validation of the International Physical Activity Questionnaire (IPAQ) for older adults: A systematic review. Aging Clin Exp Res. 2021 [cited 2024 Dec 18]. Available from: https://doi.org/10.1007/s40520-020-01578-6.

[14] Ribeiro PCC, Pires FMS, Marucci MFN, Neri AL, Pinto JM. Validação do BOMFAQ para avaliação funcional de idosos no Brasil. Rev Saúde Pública. 2011 [cited 2024 Dec 19]. Available from: https://doi.org/10.1590/S0034-89102011000500022.

[15] Almeida OP, Almeida SA. Confiabilidade da versão brasileira da Escala de Depressão em Geriatria (GDS) versão reduzida. Arq Neuropsiquiatr. 1999 [cited 2024 Dec 19]. Available from: https://doi.org/10.1590/S0004-282X1999000300013. 10.1590/s0004-282x1999000300013 10450349

[16] Boo YY, Jutila OE, Cupp MA, Manikam L, Cho SI. Korean Longitudinal Study of Aging (KLoSA): Health, Retirement, and the Role of Social Support in South Korea. Seoul [Internet]. Korea Emp Infor Ser. 2019 [cited 2024 Dec 21]. Available from: https://www.keis.or.kr/keis/en/bbs/297

[17] Smith BJ, Lim MH, Parker S. COVID-19: Vulnerability and the power of privilege in a pandemic. Health Promot J Aust. 2020; [cited 2024 Dec 21]. Available from: https://doi.org/10.1002/hpja.333. 10.1002/hpja.333 PMC7165578 32197274

[18] Stepton A, Zaninotto P. English longitudinal study of ageing: waves 0-9, 1998-2019 [Internet]. UK Data Service; 2020 [cited 2024 Dec 22]. Available from: https://doi.org/10.5255/UKDA-SN-5050-23.

[19] Moreno C, Sánchez R, González O, Pizarro R, Vargas F. Vaccine hesitancy and acceptance among older adults during the COVID-19 pandemic. Vaccine. 2021 [cited 2024 Dec 22]. Available from: https://doi.org/10.1016/j.vaccine.2021.04.073.

[20] Moyer BC, Wall HK, Carlson SA, Peng-Jun L, Greenlund KJ. Impact of the COVID-19 pandemic on routine health care services among older adults [Internet]. Natl Center Health. 2021 [cited 2024 Dec 23]. Available from: https://www.cdc.gov/nchs/products/databriefs/db400.htm.

[21] Brasil. Ministério da Saúde. Impacto da pandemia na prática de atividade física entre idosos. Boletim Epidemiológico [Internet]. Brasília: Ministério da Saúde; 2020 [cited 2024 Dec 23]. Available from: https://www.saude.gov.br/boletim-epidemiologico.

[22] Okubo Y, Kobayashi Y, Komatsu K, Miyazaki H, Tamaki T. Changes in cognitive function among older adults during the COVID-19 pandemic: A cross-sectional study in Japan. Geriatr Gerontol Int. 2021 [cited 2024 Dec 27]. Available from: https://doi.org/10.1111/ggi.14236.

[23] González-Sanguino C, Ausín B, Castellanos MA, Saiz J, López-Gómez A, Ugidos C, Muñoz M. Mental health consequences during the initial stage of the 2020 Coronavirus pandemic (COVID-19) in Spain. Psychiatry Res. 2020 [cited 2024 Dec 28]. Available from: https://doi.org/10.1016/j.psychres.2021.113089. 10.1016/j.bbi.2020.05.040 PMC7219372 32405150

[24] Di Santo SG, Franchini F, Filiputti B, Martone C, Sferrazza Papa G. The effects of COVID-19 quarantine on older adults’ cognitive function: A study in Italy. Aging Clin Exp Res. 2020 [cited 2024 Dec 28]. Available from: https://doi.org/10.1007/s40520-020-01694-3.

[25] Baker C, Brown CJ, Anderson RT, Middleton E, Meneses K, Kennedy R. Impact of COVID-19 on cognitive function in older adults: A cross-sectional study. J Geriatr Psychiatry Neurol. 2021 [cited 2024 Dec 28]. Available from: https://doi.org/10.1177/08919887211000123.

[26] Chen JJ, Liu LF, Shea JL. The Impact of Positive Self-Perceptions of Aging on Subjective Well-Being through the Mediation of Psychological Resilience among Community-Dwelling Older Adults during COVID-19 in Taiwan [Internet]. Health and Social Care in the Community. 2024 [cited 2025 Set 30]. Available from: https://doi.org/10.1155/2024/4755146

[27] Monin JK, Ali T, Syme ML, Bouldin ED, Edwards RD, Spangler R. Older adults’ experiences of loneliness and isolation during the COVID-19 pandemic: A cross-sectional study in France. Gerontologist. 2020 [cited 2024 Dec 29]. Available from: https://doi.org/10.1093/geront/gnaa105.

[28] Programa das Nações Unidas para o Desenvolvimento. Atlas do Desenvolvimento Humano no Brasil: Índice de Desenvolvimento Humano Municipal – Florianópolis [Internet]. Brasília: PNUD; 2020 [cited 2024 Dec 29]. Available from: https://www.br.undp.org.

[29] Camarano AA. Os dependentes da renda dos idosos e o coronavírus: órfãos ou novos pobres? In: Silva SP, Corseuil CH, Costa JS. (orgs.). Impactos da pandemia de COVID-19 no mercado de trabalho e na distribuição de renda no Brasil [Internet]. Brasília: Ipea; 2022 [cited 2025 Set 2]. Available from: http://doi.org/10.38116/978-65-5635-042-4/capitulo18

[30] Lima-Costa MF, Macinko J, Mambrini JVM. Hesitação vacinal contra a COVID-19 em amostra nacional de idosos brasileiros: iniciativa ELSI-COVID, março de 2021 [Internet]. Epidemiologia e Serviços de Saúde. 2022 [cited 2025 Set 10]. Available from: https://doi.org/10.1590/S1679-49742022000100020 10.1590/S1679-49742022000100020 35544866

