# Supplementary material for: EpiFloripa COVID-Tel: methods and results of the telephone survey on the COVID-19 pandemic among older adults participating in the 2017–2019 and 2021–2022 EpiFloripa cohort study
Source: Epidemiol Serv Saude. 2026 Jul 24;35:e20250021. doi: 10.1590/S2237-96222026v35e20250021.en (PMC13686413; doi:10.1590/S2237-96222026v35e20250021.en)
Supplement: Translation

# Translation: EpiFloripa COVID-Tel: métodos e resultados do inquérito telefônico sobre a pandemia de covid-19 entre pessoas idosas participantes da coorte EpiFloripa 2017-2019 e 2021-2022

**DOI:** 10.1590/S2237-96222026v35e20250021.pt

**Authors:** Eliana Funk, Danúbia Hillesheim, Thamara Hubler Figueiró, Ana Luisa Lages Belchor, Cassiano Ricardo Rech, Eleonora d’Orsi, Anna Quialheiro

**Affiliations:** 1 Universidade Federal de Santa Catarina Florianópolis Florianópolis SC Brasil Universidade Federal de Santa Catarina, Florianópolis, SC, Brasil; 2 Instituto Politécnico de Saúde do Norte Unidade de Investigação em Inteligência Artificial e Saúde Vila Nova de Famalicão BR Portugal Instituto Politécnico de Saúde do Norte, Unidade de Investigação em Inteligência Artificial e Saúde, Vila Nova de Famalicão, BR, Portugal

**Keywords:** COVID-19, Infecções por Coronavírus, Idoso, Pandemias, Inquéritos Epidemiológicos

## Abstract

**Objetivos:**

Analisar os impactos da pandemia de coronavírus nas condições de saúde e comportamentos das pessoas idosas participantes da coorte EpiFloripa Idoso, comparando as ondas 3 (2017-2019) e 4 (2021-2022), e descrever a metodologia do inquérito utilizado.

**Métodos:**

Foram realizadas entrevistas telefônicas. A coleta incluiu informações sociodemográficas, de saúde, adesão às medidas de distanciamento, uso de internet e percepção sobre a pandemia. Análises estatísticas descritivas foram realizadas para avaliar as mudanças antes e durante a pandemia. Calcularam-se medidas de frequência (n, %) e intervalos de confiança de 95%.

**Resultados:**

Utilizaram-se dados de 1.335 pessoas idosas entrevistadas na onda 3 e 354 participantes na onda 4. Houve aumento no uso de internet (de 48,5% na onda 3 para 65,5% na onda 4), o que indicou maior inclusão digital durante a pandemia. A autoavaliação de saúde mostrou que 55,6% mantiveram percepção positiva, mas 10,9% passaram a avaliá-la negativamente e 61,4% passaram a apresentar sintomas depressivos. A maioria das pessoas idosas (53,7%) seguiu rigorosamente as medidas de distanciamento social; 23,2% relataram piora na saúde. Observou-se alta adesão tanto à primeira (98,0%) quanto à segunda dose da vacina (98,8%). Contudo, 20,6% apresentaram provável declínio cognitivo durante o período da covid-19.

**Conclusão:**

Houve aumento na inclusão digital, adesão às medidas de distanciamento e vacinação. Observou-se piora na percepção de saúde, aumento de declínio cognitivo e sintomas depressivos. Estes achados destacaram a necessidade de políticas públicas direcionadas à saúde mental, à inclusão digital e ao apoio social para pessoas idosas em contextos de crise.

**Aspectos éticos**

A presente pesquisa respeitou os princípios éticos, obtendo os seguintes dados de aprovação:

**Comitês de ética em pesquisa** **Números dos pareceres**, **Datas de aprovação**

Universidade Federal de Santa Catarina 1.957.977, 9/3/2017

Universidade Federal de Santa Catarina 4.909.585, 16/8/2021

**Certificado de apresentação de apreciação ética** 16731313.0.0000.0121, 48422821.5.0000.0121

Registro do consentimento livre e esclarecido Assinado por todos os participantes antes da entrevista., Para as entrevistas por telefone, o consentimento foi obtido verbalmente.

## Introdução

A pandemia de coronavírus, oficialmente declarada pela Organização Mundial da Saúde em março de 2020, representou um desafio sem precedentes para a saúde pública global, impactando, de forma desproporcional, as populações idosas devido à sua maior vulnerabilidade a desfechos graves de saúde (1). Além do impacto direto na saúde física, a pandemia resultou em consequências psicológicas e sociais significativas para as pessoas idosas e exacerbou o isolamento social, a solidão e o risco de transtornos de saúde mental, como depressão e ansiedade (2).

Analisar as consequências da pandemia nas pessoas idosas é crucial para entender os efeitos negativos e desenvolver estratégias de mitigação para futuras crises. No Canadá e na Coreia do Sul, mais de 40% das pessoas idosas relataram piora na percepção de saúde (3,4). No Brasil, as pessoas idosas tiveram o maior aumento de sentimentos de tristeza e medo, devido ao risco de contaminação e ao isolamento social (5).

O distanciamento social, essencial para conter o vírus, isolou as pessoas idosas de redes de apoio, o que afetou o bem-estar psicológico e dificultou o acesso aos cuidados médicos e à gestão de doenças crônicas (1). A interrupção dos serviços de saúde agravou a hipertensão e diabetes, elevando o risco de complicações e hospitalizações (6).

No contexto brasileiro, os efeitos da pandemia sobre a população idosa foram agravados pelas desigualdades sociais e econômicas. Em muitas regiões, o acesso limitado a cuidados de saúde e a alta prevalência de comorbidades aumentaram o risco de complicações e de óbitos por coronavírus entre essa população (2). Além disso, a vulnerabilidade socioeconômica de muitas pessoas idosas brasileiras, que dependem de programas sociais como o Benefício de Prestação Continuada e a aposentadoria, tornou essa população ainda mais suscetível aos efeitos econômicos da pandemia, como a perda de renda familiar e o aumento dos custos com saúde (7).

Estudos longitudinais, como o EpiFloripa Idoso, são essenciais para entender o impacto da pandemia na vida das pessoas idosas, pois permitem acompanhar a evolução da saúde física, mental e social ao longo do tempo. No Brasil, a falta de dados longitudinais de qualidade dificulta a formulação de políticas de saúde pública eficazes para a população idosa (8).

Diante desses desafios, analisar os impactos da pandemia na saúde das pessoas idosas é uma prioridade para pesquisadores, profissionais de saúde e formuladores de políticas. Compreender os efeitos em saúde física, mental, acesso a serviços e bem-estar social é essencial para desenvolver intervenções e políticas que promovam o envelhecimento saudável e resiliente no pós-pandemia (1).

Este estudo buscou analisar os impactos da pandemia de coronavírus nas condições de saúde e comportamentos das pessoas idosas participantes da coorte EpiFloripa Idoso, comparando as ondas 3 (2017-2019) e 4 (2021/2022), e descrever a metodologia do inquérito telefônico utilizado durante a pandemia.

## Métodos

### Delineamento do estudo 

Trata-se de estudo longitudinal realizado com dados da coorte denominada EpiFloripa Idoso. Esta foi uma coorte de base populacional realizada em Florianópolis, Santa Catarina, com pessoas idosas de 60 anos ou mais.

### Contexto e participantes

A coorte EpiFloripa Idoso foi iniciada em 2009 com residentes da área urbana de Florianópolis, com o objetivo de investigar as condições de vida e saúde da população idosa ao longo do tempo. As coletas foram realizadas em ondas de entrevistas domiciliares periódicas, sendo a primeira em 2009-2010, a segunda em 2013-2014 e a terceira em 2017-2019. Durante a pandemia, foi conduzida a quarta onda (2021-2022), cujos dados foram utilizados neste estudo. Detalhes do estudo podem ser acessados em: https://epifloripaidoso.paginas.ufsc.br/.

A terceira onda, ocorrida entre 2017 e 2019, teve seu plano amostral, os procedimentos operacionais das entrevistas domiciliares e as estratégias metodológicas previamente documentados (9). A quarta onda foi denominada EpiFloripa COVID-Tel e conduzida durante a pandemia por meio de entrevistas telefônicas, com o objetivo específico de avaliar os impactos da covid-19 na saúde, nas condições de vida e nos comportamentos dessa população idosa. O desenho longitudinal do estudo permitiu a comparação dos dados coletados antes (onda 3) e durante (onda 4) o período pandêmico.

Na onda 4, foram considerados todos os participantes da onda 3, tanto aqueles do acompanhamento quanto aqueles considerados como perda ou recusa na onda 3. Entre novembro de 2021 e novembro de 2022, foram realizadas tentativas de contato telefônico com todos os elegíveis da onda 3, sendo o período de realização da coleta. 

Entre os critérios de exclusão do EpiFloripa COVID-Tel, estavam: as perdas e recusas dos participantes do EpiFloripa Adulto que foram convidados a participar da pesquisa na terceira onda; e os novos participantes selecionados que recusaram a realização das entrevistas domiciliares nessa fase, não disponibilizando, portanto, informações para este estudo. O participante que, em razão de comprometimento cognitivo ou auditivo, não pôde responder ao questionário por telefone, pôde autorizar um informante a fornecer as respostas em seu nome para seções específicas da entrevista.

As entrevistas remotas permitiram o acompanhamento das pessoas idosas, vulneráveis ao coronavírus, sem risco de contágio. Essa adaptação avaliou os impactos do distanciamento social na saúde. O método seguiu o Estudo Longitudinal de Saúde do Adulto, que usou entrevistas e questionários adaptados, assegurando a continuidade do estudo (8).

### Entrevista telefônica EpiFloripa COVID-Tel 

O convite para entrevistadores foi direcionado a estudantes de graduação e pós-graduação em saúde da Universidade Federal de Santa Catarina. Após o recrutamento, houve capacitação sobre o projeto e treinamento em ligações e testes. Todos foram instruídos sobre o fluxo das ligações e receberam um manual. Os aptos receberam listas de contatos das pessoas idosas para o agendamento e a realização das entrevistas.

As pessoas idosas elegíveis foram contatadas por telefones fixos, móveis ou por contatos de amigos e familiares do banco de dados do EpiFloripa Idoso. O entrevistador se identificava, e, após o consentimento oral, a entrevista prosseguia. Caso a pessoa idosa estivesse indisponível, a ligação era reagendada.

O questionário do estudo abordou questões como dados sociodemográficos e aspectos relacionados com a pandemia, como adesão às normas de distanciamento físico, uso de máscara, vacinação, positividade do teste para coronavírus e internação por coronavírus. Todos os instrumentos de coleta de dados utilizados no inquérito consistiram em questionários previamente validados e amplamente utilizados em estudos epidemiológicos, garantindo a confiabilidade e validade das medidas investigadas. O questionário completo está disponível em: https://epifloripaidoso.paginas.ufsc.br/.

O questionário foi transcrito com o uso da ferramenta Research Electronic Data Capture v9.1.0. O controle de qualidade foi realizado por meio de ligações telefônicas para uma subamostra de 10% dos participantes.

### Perdas, recusas e óbitos

Os óbitos foram identificados durante as chamadas telefônicas. Quando um óbito era informado, solicitavam-se dados sobre a data, a causa e o local do falecimento para registro na base de dados. As recusas foram expressas verbalmente, enquanto as perdas foram caracterizadas após três tentativas de contato realizadas em dias e horários distintos, incluindo finais de semana.

### Variáveis e mensuração

Este estudo utilizou as seguintes variáveis: sexo (masculino, feminino), faixa etária (60-69, 70-79, 80 anos ou mais), escolaridade (1-4, 5-8, 9-11, 12 anos ou mais), estado civil (casado[a]/com companheiro[a], solteiro[a], divorciado[a], viúvo[a]), cuidador (sim, não), renda per capita (≤1,0; 1,^1^-^5^,0; 5,^1^-^10^,0; ≥10,0 salários mínimos) e uso de internet (sim, não).

O déficit cognitivo foi avaliado pelo Mini Exame do Estado Mental na onda 3, classificado segundo Almeida (10), e pelo instrumento Avaliação Cognitiva de Montreal (11) adaptado para entrevista por telefone na onda 4, categorizado em sem déficit cognitivo e provável déficit. A pontuação proposta por Trzepacz (12) foi utilizada para equiparação dos instrumentos. A atividade física foi medida pelo Questionário Internacional de Atividade Física (13), validado para pessoas idosas, e classificada como inativo (0-9 min/sem), insuficientemente ativo (10-149 min/sem) e ativo (≥150 min/sem). 

As atividades de vida diária foram avaliadas em ambas as ondas pelo Questionário Brasileiro de Avaliação Funcional Multidimensional (14), categorizadas em independente, dependente leve e dependente moderado ou grave. A autoavaliação de saúde foi classificada como positiva ou negativa. A suspeita de depressão foi avaliada pela Escala Geriátrica de Depressão (15), categorizada em sem sintomas depressivos e com sintomas depressivos, segundo Almeida (15).

As variáveis sobre a pandemia foram compostas de nove perguntas. A variável “Nos primeiros seis meses de pandemia, como o(a) Sr.(a) agiu diante das restrições?” foi categórica nominal, com as opções: “não fez nada”, “tomou cuidados, mas continuou saindo”, “ficou em casa, saindo só para compras” e “ficou rigorosamente em casa”. O estudo incluiu as variáveis percepção de saúde (“ficou igual”, “melhorou”, “piorou um pouco”, “piorou muito”), mudanças na renda (“aumentou”, “ficou igual”, “diminuiu pouco”, “diminuiu muito”, “ficou sem renda”) e frequência de uso de espaços públicos (“nunca”, “todos os dias”, “2-3 dias na semana”, “1 vez na semana”, “2-3 vezes no mês”, “1 vez por mês”). Internação hospitalar, realização do teste da covid-19, vacinação (1^a^ e 2^a^ dose) e uso de farmácia foram avaliadas como variáveis dicotômicas (sim, não).

### Métodos estatísticos

Os dados foram analisados por meio de estatística descritiva para caracterização da amostra das pessoas idosas das ondas 3 e 4, com uso do comando “prop” para ajustar o viés amostral, e intervalos de confiança de 95%. As frequências absolutas e relativas dos dados foram apresentadas. 

Para a apresentação das variáveis de mudança, novas variáveis politômicas foram criadas a partir das variáveis dicotômicas nos momentos onda 3 e onda 4. Essas novas variáveis foram codificadas em: 1=continuou a ter a condição (1-1); 2=deixou de ter a condição (1-0); 3=continuou a não ter a condição (0-0); e 4=passou a ter a condição (0-1). 

As análises foram realizadas no software estatístico Stata, versão 18.5, MP-Parallel edition.

## Resultados

Os resultados apresentados referem-se a dois períodos distintos do estudo: a onda 3, conduzida entre 2017 e 2019, na qual 1.335 pessoas idosas foram entrevistadas presencialmente, e a onda 4, realizada entre 2021 e 2022. Para a execução da onda 4, foram identificados os indivíduos elegíveis provenientes das ondas anteriores, o que totalizou 1.646 pessoas idosas. Entre estas, 363 participaram do estudo EpiFloripa COVID-Tel (onda 4), sendo 354 entrevistas completas e 9 incompletas – estas últimas interrompidas a pedido do participante durante a ligação telefônica. Assim, foram consideradas válidas as 354 entrevistas completas realizadas por telefone, o que correspondeu a 21,5% do total de elegíveis. As perdas de seguimento ocorreram em decorrência de óbitos (n=74), recusas (n=274) e contatos telefônicos não concluídos após 3 tentativas ou números inválidos (n=935) (Figura 1).

**Figura 1:** Onda 4 do estudo de coorte EpiFloripa Idoso. Florianópolis, 2021-2022 (n=354)

Apresentaram-se as características sociodemográficas e de saúde das pessoas idosas nas ondas 3 e 4 do estudo (Tabela 1). Houve maior frequência do sexo feminino e envelhecimento da amostra, com maior proporção de pessoas idosas acima de 80 anos na onda 4 (31,0%). Houve aumento no uso da internet entre as pessoas idosas, passando de 48,5% para 65,5%, além de crescimento na proporção daquelas com provável declínio cognitivo (20,6%). A inatividade física permaneceu elevada (47,4%). Observou-se também maior nível de escolaridade, uma vez que 42,0% das pessoas idosas apresentaram 12 anos ou mais de estudo, e estabilidade da renda familiar ao longo do período analisado (Tabela 1).

**Tabela 1:** Frequência relativa (%) e intervalo de confiança de 95% (IC95%), segundo características sociodemográficas: onda 3 (pré-covid-19) e onda 4 (durante a covid-19). Florianópolis, 2017-2022

Variável	Onda 3 (n=1.335) % (IC95%)	Onda 4 (n=354) % (IC95%)
Sexo		
Feminino	61,0 (58,3; 63,6)	64,1 (58,9; 68,9)
Masculino	39,0 (36,4; 41,7)	35,9 (31,0; 41,0)
**Faixa etária **(anos)		
60-69	34,6 (32,0; 37,1)	18,4 (14,7; 22,8)
70-79	41,5 (38,9; 44,2)	50,6 (45,4; 55,8)
80+	23,9 (21,8; 26,3)	31,0 (26,5; 36,1)
**Escolaridade **(anos)		
Sem escolaridade	4,7 (3,7; 5,9)	1,9 (0,8; 4,0)
1-4	28,8 (26,4; 31,3)	21,6 (17,5; 26,4)
5-8	17,0 (15,1; 19,1)	16,8 (13,2; 21,2)
9-11	17,1 (15,2; 19,3)	17,7 (13,9; 22,2)
12+	32,4 (29,9; 34,9)	42,0 (36,8; 47,4)
**Estado civil**		
Casado(a)/companheiro(a)	55,4 (52,7; 58,0)	53,4 (48,2; 58,5)
Solteiro(a)	6,6 (5,4; 8,1)	4,8 (2,8; 7,3)
Divorciado(a)/separado(a)	11,5 (9,9; 13,4)	7,1 (4,8; 10,3)
Viúvo(a)	26,5 (24,2; 29,0)	34,7 (30,7; 38,9)
Cuidador		
Sim	10,0 (8,5; 11,8)	9,1 (6,2; 12,2)
Não	90,0 (88,2; 91,8)	90,9 (87,5; 93,5)
**Renda per capita **(**salário mínimo**)		
≤1,0	25,5 (23,2; 27,9)	21,6 (17,5; 26,4)
1,1-5,0	57,5 (54,8; 60,1)	57,4 (51,9; 62,6)
5,1-10,0	12,4 (10,8; 14,3)	15,0 (11,6; 19,3)
10,0+	4,6 (3,6; 5,8)	6,0 (3,9; 9,1)
**Uso de internet**		
Sim	48,5 (45,9; 51,2)	65,5 (60,4; 70,3)
Não	51,5 (48,8; 54,1)	34,5 (29,7; 39,6)
**Déficit cognitivo**		
Sem déficit cognitivo	80,5 (78,3; 82,5)	79,4 (74,8; 83,3)
Provável déficit	19,5 (17,5; 21,7)	20,6 (16,7; 25,2)
**Atividade física **(minutos/semana)		
Inativo (0-9)	54,5 (51,8; 57,2)	47,4 (42,3; 52,7)
Insuficientemente ativo (10-149)	18,6 (16,6; 20,8)	22,7 (18,6; 27,4)
Ativo (≥150)	26,9 (24,6; 29,3)	29,9 (25,3; 34,8)
**^B^OMFAQa **(AVD)^b^		
Independente	30,8 (28,3; 33,3)	41,2 (36,1; 46,5)
Dependente leve	35,9 (33,4; 38,6)	27,4 (22,9; 32,3)
Dependente moderado ou grave	33,3 (30,8; 35,9)	31,4 (26,7; 36,5)
**Autoavaliação da saúde**		
Positiva	61,9 (59,3; 64,6)	66,7 (61,2; 71,7)
Negativa	38,1 (35,4; 40,7)	33,3 (28,3; 38,8)

^a^BOMFAQ: Brazilian Older Americans Resources and Services Multidimensional Function Assessment Questionnaire; ^b^AVD: atividades da vida diária.

As medidas de distanciamento social foram seguidas rigorosamente por 53,7% dos participantes, com 1,1% dos casos de internação por covid-19. A percepção de piora na saúde foi de 23,2% (piorou um pouco e piorou muito), e o uso de espaços públicos como praças e parques foi reduzido (65,8% nunca frequentaram). Os dados indicaram alta adesão à vacinação, visto que 98,8% dos participantes tinham recebido as duas doses recomendadas (Tabela 2).

**Tabela 2:** Frequências absoluta (n) e relativa (%) referente às questões sobre o comportamento das pessoas idosas e consequências para estas durante a covid-19 (onda 4). Florianópolis, 2021-2022 (n=354)

Variável	Onda 4 n (%)
**Nos primeiros seis meses de pandemia, como o(a) Sr.(a) agiu diante das restrições**?	
Não fez nada, levou a vida normal	12 (3,4)
Procurou tomar cuidados, ficar à distância das pessoas, não visitar outras pessoas, mas continuou trabalhando e/ou saindo	49 (13,8)
Ficou em casa, só saindo para compras	101 (28,5)
Ficou rigorosamente em casa, saindo só por necessidades de atendimento à saúde	190 (53,7)
Não sabe/não quer informar	2 (0,6)
**Durante a pandemia, o(a) Sr.(a) precisou sair para ir à farmácia**?	
Sim	46 (13,0)
Não	306 (86,4)
Não sabe/não quer informar	2 (0,6)
**O(a) Sr.(a) acha que a pandemia provocou mudanças no seu estado de saúde**?	
Ficou igual	263 (74,3)
Melhorou	5 (1,4)
Piorou um pouco	59 (16,7)
Piorou muito	23 (6,5)
Não sabe/não quer informar	4 (1,1)
**Nos últimos três meses, durante a pandemia, com que frequência o(a) Sr.(a) utilizou algum parque/praça/orla marítima próximo de sua residência**?	
Nunca	233 (65,8)
Todos os dias da semana	12 (3,4)
2-3 dias por semana	44 (12,4)
1 vez por semana	18 (5,1)
2-3 vezes por semana	15 (4,2)
1 vez por mês	20 (5,7)
Não sabe/não quer informar	12 (3,4)
**O(a) Sr.(a) fez o teste para saber se estava infectado(a) pelo vírus da covid**-19?	
Sim	149 (42,1)
Não	205 (57,9)
**O(a) Sr.(a) precisou de internação por conta do novo coronavírus**?	
Sim	4 (1,1)
Não	350 (98,9)
**Como a pandemia afetou a renda da família**?	
Aumentou	7 (2,0)
Foi mantida igual	263 (74,3)
Diminuiu um pouco	53 (15,0)
Diminuiu muito	23 (6,5)
Ficou sem renda	1 (0,2)
Não sabe/não quer informar	7 (2,0)
**O(a) Sr.(a) tomou a primeira dose da vacina contra a covid**-19?	
Sim	347 (98,0)
Não	7 (2,0)
**O(a) Sr.(a) tomou a segunda dose da vacina contra a covid**-19?	
Sim	343 (98,8)
Não	3 (0,9)
Não se aplica, tomou dose única	1 (0,3)

Destacaram-se as mudanças nas condições de saúde e funcionais entre as ondas 3 e 4 (Figura 2). Observou-se que 13,5% das pessoas idosas passaram a ter declínio cognitivo e 61,4% passaram a ter sintomas de depressão durante a pandemia. A autoavaliação de saúde mostrou que 55,6% mantiveram percepção positiva (Figura 2).

**Figura 2:** Frequência relativa (%) referente às mudanças nos desfechos de saúde entre a onda 3 (2017-2019) e onda 4 (2021-2022). Florianópolis, 2017-2022 (n=354)

## Discussão

Os resultados deste estudo evidenciaram os principais impactos da pandemia de covid-19 na saúde e nos comportamentos de pessoas idosas da coorte EpiFloripa. De modo geral, observou-se que, embora muitas tenham mantido percepções positivas de saúde e adotado medidas preventivas, houve importantes efeitos adversos em dimensões cognitivas, emocionais e físicas. A inclusão digital destacou-se como fator adaptativo durante o distanciamento social, enquanto a alta adesão à vacinação refletiu a confiança nas medidas de saúde pública.

Com relação ao envelhecimento da coorte, a maior proporção de participantes com 80 anos ou mais na onda 4 esteve em consonância com o esperado em estudos longitudinais, que refletiram mortalidade diferencial e alterações na composição etária ao longo do tempo (8). A maior proporção de viúvos na onda 4 (34%) em comparação à onda 3 (26%) corroborou a literatura que associa envelhecimento com aumento da viuvez, especialmente entre mulheres, e seus efeitos no bem-estar emocional (16,17).

Além do aumento absoluto na prevalência do uso de internet (de 48% para 65%), os dados sugeriram avanços em inclusão digital entre as pessoas idosas ao longo do período, possivelmente impulsionados pelas demandas de comunicação, acesso à informação e serviços durante a pandemia. O uso de tecnologias digitais favoreceu o acesso a informações, serviços de saúde e interações sociais remotas durante a pandemia (18,19). No entanto, em média, 34% das pessoas idosas permaneceram sem acesso à internet, o que pode estar relacionado a barreiras socioeconômicas e educacionais (20). Essas desigualdades tecnológicas indicaram a necessidade de políticas públicas voltadas à alfabetização digital para pessoas idosas (21,22).

Os resultados também revelaram aumento na prevalência de provável declínio cognitivo (20%) e sintomas depressivos (61%). O impacto do isolamento social, a interrupção de rotinas e a ausência de estímulos cognitivos estão entre os principais fatores associados a esse declínio (23). Estudos internacionais corroboraram esses achados: na Itália, 30% das pessoas idosas relataram piora da memória e atenção (24); nos Estados Unidos, cerca de 24% dos idosos referiram piora subjetiva da função cognitiva na pandemia, em um contexto marcado por alterações na rotina e menor engajamento em atividades cognitivamente estimulantes (25); na América Latina, entre 20% e 25% das pessoas idosas apresentaram comprometimento cognitivo, com maior prevalência entre os menos escolarizados (6).

Em relação à saúde mental, 61,4% dos participantes apresentaram suspeita de depressão na onda 4, resultado alarmante quando comparado a períodos pré-pandêmicos. Identificaram-se sentimentos como a solidão, o luto e o medo da contaminação como gatilhos principais do agravamento dos sintomas (3,17). Na Espanha, houve aumento da ansiedade e depressão em 40% das pessoas idosas durante a pandemia (23), enquanto no Brasil também houve alta prevalência de sofrimento psíquico entre pessoas idosas (2,5).

A redução da prática de atividade física foi expressiva: 47% das pessoas idosas foram classificadas como inativas e 22% como insuficientemente ativas. Este achado é compatível com evidências do Canadá e do Brasil, que indicaram diminuição de até 60% na atividade física de pessoas idosas em decorrência do fechamento de espaços públicos e da adoção do distanciamento social (20,21). Tal redução contribuiu para a piora da saúde funcional e para o aumento do risco de doenças crônicas (21).

A autoavaliação negativa de saúde, relatada por 10% dos participantes, esteve associada ao isolamento social e à restrição de atividades. Achados semelhantes foram observados em um estudo na Coréia (4), no qual 20% dos 2.097 participantes relataram piora no estado de saúde pós-pandemia, e em Taiwan (26), onde percepções negativas de bem-estar subjetivo durante a pandemia correlacionaram-se com piores autoavaliações de saúde entre pessoas idosas. Por outro lado, parte das pessoas idosas relatou melhora ou manutenção da percepção positiva, sugerindo a influência de fatores como ausência de doenças graves e suporte familiar, e da percepção comparativa diante do contexto vivido por outros (26).

Quanto à adesão às medidas sanitárias, 53% das pessoas idosas permaneceram rigorosamente em casa, o que coincidiu com o cenário da Itália e da França (25,27). Essa adesão contribuiu para a baixa frequência de internação (1%) observada na onda 4. A maioria também evitou saídas para farmácias (86%), o que indicou adaptação ao uso de entregas e apoio de redes sociais próximas (27).

A estabilidade da renda familiar relatada por 74% dos participantes pôde estar relacionada à estrutura socioeconômica local, ao índice de desenvolvimento humano elevado de Florianópolis (0,847) e à presença de aposentadorias estáveis (28). Isso contrastou com estudos em regiões menos favorecidas, onde a pandemia teve maior impacto econômico sobre as pessoas idosas (7,29).

A adesão à vacinação foi elevada: 98% das pessoas idosas tomaram a primeira dose, e 98% destas, a segunda. Os achados refletiram o êxito das campanhas de imunização contra a covid-19 entre pessoas idosas no Brasil, corroborado por evidências de outro estudo nacional, no qual, entre 4.364 participantes, 91% pretendiam se vacinar ou já haviam sido vacinados (30).

Este estudo apresentou limitações relevantes. O uso de autorrelato pode introduzir viés de memória e resposta, particularmente em variáveis subjetivas. A subamostra da onda 4, composta por 354 participantes, não é representativa da coorte original, e o viés de sobrevivência pode ter influenciado os resultados, considerando que as pessoas idosas mais saudáveis tenderam a permanecer na amostra. A taxa de perdas entre as ondas é comparável a outros estudos longitudinais com pessoas idosas (6,8), mas limita inferências generalizáveis. Além disso, a coleta telefônica, embora necessária, pode ter aumentado as recusas, em função de desconfianças com esse tipo de abordagem (7).

Apesar das limitações, estas foram minimizadas por estratégias metodológicas como o treinamento dos entrevistadores, a utilização de instrumentos previamente validados e adaptados para aplicação por telefone, o uso de manuais padronizados e a realização do controle de qualidade por meio da reaplicação em subamostra. Isso assegurou maior confiabilidade aos dados coletados. 

Os resultados da coorte EpiFloripa evidenciaram que, embora as pessoas idosas tenham adotado estratégias de adaptação relevantes durante a pandemia, os efeitos adversos sobre a saúde mental, cognitiva e física foram importantes. Tais evidências reforçaram a necessidade de políticas públicas contínuas e específicas para a população idosa em contextos de crise sanitária.

Este estudo representou uma das poucas análises longitudinais realizadas no Brasil sobre os impactos da pandemia na população idosa. Ofereceram-se evidências valiosas para subsidiar políticas intersetoriais que promovam inclusão digital, apoio à saúde mental e acesso contínuo aos cuidados de saúde, com foco na promoção do envelhecimento mais saudável.

## Data Availability

O banco de dados e os códigos de análise utilizados na pesquisa estarão disponíveis sob demanda dos pareceristas.
